# Antibiotic resistance pattern, capsular types, and molecular characterization of invasive isolates of *Streptococcus pneumoniae* in the south of Tunisia from 2012 to 2018

**DOI:** 10.1186/s12866-023-02784-2

**Published:** 2023-02-04

**Authors:** Sonia Ktari, Nourelhouda Ben Ayed, Imen Ben Rbeh, Nourhène Garbi, Sonda Maalej, Basma Mnif, Faouzia Rhimi, Adnene Hammami

**Affiliations:** 1 Laboratory of Microbiology, Faculty of Medicine Sfax, University of Sfax-Tunisia, Avenue Majida Boulila, 3027 Sfax, Tunisia; 2 Research Laboratory Microorganisms and Human Disease “MPH LR03SP03”, Sfax, Tunisia; 3 Laboratory of Microbiology, Habib Bourguiba University Hospital, Sfax, Tunisia; 4 Medical Genetic Department, HediChaker Hospital, Sfax, Tunisia

**Keywords:** Invasive pneumococcal disease, Serotype, Antibiotic resistance, Clonal complex, MLST

## Abstract

**Background:**

*Streptococcus pneumoniae* remains a leading cause of morbidity and mortality worldwide. In this study, we sought to analyze serotype distributions, antibiotic resistance, and genetic relationships of 106 clinical invasive pneumococcal isolates recovered in Tunisia between 2012 and 2018, prior to the routine use of pneumococcal conjugate vaccines (PCV).

**Methods:**

We used multiplex PCR, the disk diffusion method and/or E-test, and multi-locus sequence typing (MLST).

**Results:**

The most frequent serotypes were 14 (17%), 19F (14.2%), and 3 (11.3%). Of the 106 *S. pneumoniae* isolates, 67.9% were penicillin non-susceptible (29.4% were resistant), 45.3% were amoxicillin non-susceptible (17% were resistant), and 16% were cefotaxime non-susceptible. For antibiotics other than β-lactams, resistance rates to erythromycin, tetracycline, cotrimoxazole, and chloramphenicol were 62.3, 33, 22.6, and 4.7%, respectively. Two isolates were non-susceptible to levofloxacin. Among 66 erythromycin-resistant pneumococci, 77.3% exhibited the cMLSB phenotype, and 87.9% carried *erm*B gene. All tetracycline-resistant strains harbored the *tet*M gene. The potential coverage by 7-, 10-, and 13-valent pneumococcal conjugate vaccines were 55.7, 57.5, and 81.1%, respectively. A multilocus sequence typing analysis revealed great diversity. Fifty different sequence types (STs) were identified. These STs were assigned to 10 clonal complexes and 32 singletons. The most common STs were 179, 2918, 386, and 3772 – related mainly to 19F, 14, 6B/C, and 19A serotypes, respectively.

**Conclusions:**

This study demonstrated that the majority of the serotypes of invasive pneumococci in the Tunisian population were 14, 19F, and 3. Moreover, we noted a high degree of genetic diversity among invasive *S. pneumoniae* isolates. The highest proportions of antibiotic non-susceptible isolates were for penicillin, erythromycin, and tetracycline. Further molecular characteristics are required to monitor the genetic variations and to follow the emergence of resistant pneumococci for the post-vaccination era in Tunisia.

## Introduction

*Streptococcus pneumoniae* (*S. pneumoniae*) is an important pathogen causing invasive diseases such as sepsis, meningitis, and pneumonia worldwide and contributing to significant morbidity and mortality, particularly in young children and the elderly [1, 2]. In 2010, The European Surveillance System reported an overall incidence of 5.2 cases of invasive pneumococcal disease (IPD) per 100,000 population, with the most affected age groups being < 1 year and ≥ 65 years old [3]. Antibiotics and vaccines are strategies currently available to fight against pneumococcal infections and to prevent them. However, since the late 1970s, *S. pneumoniae* resistant to penicillin and other antibiotics, such as macrolides, has emerged and rapidly increased worldwide, mostly related to the misuse of these drugs in respiratory infections [4–6]. Based on capsular polysaccharide composition, almost 100 serotypes of *S. pneumoniae* have been identified [7]. However, some serotypes are associated with IPD [1, 8–11]. Moreover, vaccine serotypes, such as 6B, 9 V, 14, 19A, 19F, and 23F, are more likely than others to be resistant to antimicrobials [9, 12]. In Tunisia, the highest prevalence of non-susceptible *S. pneumoniae* was reported for penicillin and macrolide [13–16]. Pneumococcal conjugate vaccines (PCVs) remain the most effective strategies to reduce invasive pneumococcal infections [17, 18]. Since April 2019, PCV10 has been incorporated into the national immunization program for childhood vaccination. Despite the availability of PCVs in several countries around the world, the reduction in PCV-type IPD has been offset by the expansion of non-vaccine serotypes. The replacement of vaccines with non-vaccine serotypes is mostly associated with the emergence of multidrug-resistant serotypes [9, 12].

To monitor the epidemiology of *S. pneumoniae*, several molecular typing methods were developed [19]. Of these, multilocus sequence typing (MLST) can provide good resolving and discriminatory power that can be used for local and global epidemiology [19]. MLST might provide important information on evolutionary changes, such as the expansion of existing serotypes or clones, the emergence of new clones, or capsular switching. MLST remains a very useful molecular method to oversee the spread of national and international clones through the pneumococcal population. Based on MLST, Pneumococcal Molecular Epidemiology Network (PMEN) published 43 international clones of *S. pneumoniae* associated particularly with the most common resistant serotypes, namely 6A, 6B, 14, 15A, 19A, 19F, 23F, and 35B. Despite the expensive cost of the MLST method, this technique makes it possible to share data, and the allelic profiles can easily be compared to those available in MLST Database.

In the present study, we sought to investigate the antibiotic resistance pattern, capsular types, and molecular characterization of invasive *S. pneumoniae* isolates in Sfax, the south of Tunisia, over a seven-year period (2012- 2018).

## Materials and methods

### Bacterial isolates

We reported a retrospective study including a total of 106 clinical invasive *S. pneumoniae* isolates collected between 2012 and 2018 at the microbiology laboratory of Habib Bourguiba University Hospital, Sfax, Tunisia. These isolates were identified by standard procedures, including Gram staining, optochin sensitivity, and bile solubility tests. All isolates were *cps*A gene positive by PCR amplification.

### Antibiotic susceptibility testing

Antimicrobial susceptibility testing was performed using the antibiogram method on 5% horse blood-enriched Mueller-Hinton agar according to the standardization technique of the Antibiogram Committee of the French Society for Microbiology and the European Committee on Antimicrobial Susceptibility Testing (CA-SFM/EUCAST, 2018). All isolates were tested for penicillin, amoxicillin, cefotaxime, tetracycline, chloramphenicol, erythromycin, lincomycin, levofloxacin, rifampicin, trimethoprim-sulfamethoxazole, vancomycin, and teicoplanin. Minimum inhibitory concentrations (MICs) of penicillin, amoxicillin, cefotaxime, and levofloxacin were determined by the E-test method. CA-SFM/EUCAST, 2018 breakpoints were applied for all isolates. Oxacillin (1 μg) disk diffusion testing was performed for the prediction of penicillin-resistant pneumococci [20]. The double disk diffusion method with erythromycin (15 μg) and clindamycin (2 μg) disks were used to differentiate constitutive and inducible macrolide resistance phenotype [21]. An internal quality control was performed using *S. pneumoniae* ATCC49619.

### Detection of resistance genes

The PCR assays were used to detect erythromycin and tetracycline genes including *erm*B, *mef* A/E, *tet*M, *tet*O, and transposon-related genes [22–25]. The PCR primers used are shown in Table 1. To distinguish the *mef*A and *mef*E gene subclasses, the PCR products of the *mef* gene were digested with *Bam*HI and *Nhe*I, instead of *Dra*I [23].

**Table 1:** The primer sets used to detect erythromycin and tetracycline genes

Target	Primer sequence	Reference
*erm*B	5′-GAAAAAGTACTCAACCAAATA-3′ 5′-AGTAATGGTACTTAAATTGTTTAC-3′	[22]
*mef*A/E	5′-GCGTTTAAGATAAGCTGGCA-3′ 5′-CCTGCACCATTTGCTCCTAC-3′	[23]
*tet*M	5′-AGTTTTAGCTCATGTTGATG-3′ 5′-TCCGACTATTTGGACGACGG-3′	[24]
*tet*O	5′-ACGGARAGTTTATTGTATACC-3′ 5′-TGGCGTATCTATAATGTTGAC-3′	[25]

### Capsular typing

Capsular typing was performed with a combination of multiplex PCRs targeting serotypes/serogroups as described previously [15, 26]. Next, depending on the amplification pattern obtained, a simplex PCR reaction was performed for each serotype. Strains that could not be serotyped by multiplex PCR reaction were serotyped using a conventional PCR targeting other serotypes with primers as described previously [27]. One representative of each serotype was confirmed by sequence analysis. Isolates determined as 6A/B or 9 V/A (vaccine serotypes) by the multiplex PCR method were typed to the serotype level using pneumococcal capsule-specific antisera (ImmuLex™ Pneumotest).

### Multilocus sequence typing (MLST)

Seven housekeeping genes (*aro*E, *gdh*, *gki*, *rec*P, *spi*, *xpt*, and *ddl*) were amplified, sequenced, and analyzed. Alleles and sequence types (STs) were determined according to the PubMLST database (https://pubmlst.org/spneumoniae/). Sequences and STs that could not be found in the database were submitted to the curator of the database. STs were compared with the contents of the PMEN database (https://www.pneumogen.net/pmen/index.html) to identify commonly circulating clones. STs that shared at least six of seven allelic variants composed clonal complexes (CC) or groups. We confirmed the occurrence of serotype switching events when isolates sharing the same ST belonged to different serotypes. A minimum spanning tree (MST) based on MLST was built using PHYLOVIZ 2.0 [28].

### Statistical analysis and diversity power

Statistical comparisons were made using the Chi-square test or Fisher’s exact test. SPSS version 17.0 (SPSS Inc., Chicago, IL, USA) was used for the statistical analyses. *P*-values of < 0.05 were considered to be statistically significant.

The discriminatory power of the MLST subtyping method was calculated using Simpson’s diversity index (SID) with the online tool available at http://www.comparingpartitions.info.

## Results

### Bacterial collection

The 106 invasive *S. pneumoniae* isolates collected between 2012 and 2018 were recovered from blood cultures (53.8%), cerebrospinal fluid (CSF, 29.2%), puncture fluid (9.4%), deep pus and abscesses (3.8% for each). CSF samples accounted for 42.5% (17/40) in children < 5 years, of whom 16 (40%) were < 2 years, 55.5% (5/9) in older children 5–17 years, and 17.6% (9/51) in adults ≥18 years.

### Antimicrobial susceptibility and resistance genes

Of the 106 invasive isolates, 72 (67.9%) were penicillin non-susceptible. For meningococcal isolates, and based on meningitis breakpoints, 67.7% of isolates (21/31) had MICs greater than 0.06 mg/mL for penicillin, 41.9% of isolates (*n* = 13) were amoxicillin resistant with MICs greater than 0.5 mg/ml, 16.1% were non-susceptible to cefotaxime, and all isolates were susceptible to levofloxacin. For non-meningococcal isolates, and considering non-meningitis breakpoints, 68% of these isolates were penicillin non-susceptible with 19.6% of a high level of resistance (MIC = 3–12 μg/ml), 45.3 and 16% of isolates were non-susceptible to amoxicillin and cefotaxime, respectively, and two isolates were non-susceptible to levofloxacin (MIC = 1-4 μg/ml). A high level of resistance to amoxicillin was observed in 8% of isolates (MIC = 3-6 μg/ml).

For other tested antibiotics, resistance to erythromycin, tetracycline, cotrimoxazole, and chloramphenicol showed fewer frequencies in isolates from meningitis (54.8, 29, 9.7and 3.2%, respectively) than in isolates from non-meningitis infections (65.3, 34.7, 28 and 5.3%, respectively). All tetracycline-resistant isolates were *tet*M gene positive. Analyses of erythromycin-resistant invasive strains revealed that 89.4% of isolates were macrolide lincosamide-streptogramin B (MLSB) resistance phenotype with a predominance of high-level MLSB constitutive phenotype (77.3%). These isolates harbored the *erm*B gene alone. Macrolide (M) resistance phenotype was observed in 10.6% of isolates and harbored the *mef* gene alone. All *mef*-positive isolates carried the *mefE* gene. The association of *ermB* and *mefE* genes has been identified only in one isolate (Table 2). The major transposon carrying the erythromycin and tetracycline resistance genes was Tn*1545* (76.6%) followed by Tn*2009* (10.9%), Tn*6002* (7.8%), Tn*6003* (3.1%), and Tn*2010* (1.6%) (Table 2).

**Table 2:** Characteristics of invasive *S. pneumoniae* isolates with respect to their serotype, MLST profile, resistance to Penicillin, Cefotaxim Tetracyclin and Erythromycin, the source of isolation and transposon types

Serotypes (N°of isolates)	Predicted ST/CC (N°of isolates)	Associated PMEN clone	Year ()^**a**^	PeniG^**b**^ ()^**a**^	Ctx^**c**^ ()^**a**^	Tet^**d**^ ()^**a**^	Ery^**e**^ ()^**a**^	Source of isolation ()^**a**^	MLSB ()^**a**^	***ermB/mefE*** ()^**a**^	Transposon ()^**a**^
14 (18)	2918 (11)	Spain^9V^-3, SLV^j^ ST156	2013 (2) 2014 (1) 2015 (1) 2016 (4) 2017 (2) 2018 (1)	I^h^ (9) R (2)	I (4) S (7)	S^g^ (9) R (2)	R^i^(10) S(1)	Blood (6) CSF^f^ (3) Puncture fluid (1)	MLSBc (10)	*erm*B (10)	Tn*1545* (10)
	143 (3)	Spain^9 V^-3 DLV^k^ ST156	2012 (1) 2016 (1) 2018 (1)	I (1) R (2)	I (2) S (1)	I (1) S (2)	R (3)	Blood (2) Puncture fluid (1)	MLSBc (1) M (2)	*erm*B (1) *mef* E (2)	Tn*1545* (1) Tn*2009* (2)
	4949 (1)	Spain^9V^-3, DLV ST156	2014	R	S	S	R	Blood	MLSBc	*erm*B	Tn*1545*
	63 (1)		2012	I	S	R	S	CSF			
	156 (1)	Spain^9 V^-3	2015	I	S	S	S	Blood			
	4344 (1)		2015	I	S	S	S	Blood			
19F (15)	179 (13)	Portugal^19F^21, SLV ST177	2012 (2) 2013 (6) 2014 (2) 2015 (1) 2017 (2)	I (9) R (4)	I (6) S(7)	R (8) I (2) S (3)	R(12) S (1)	Abscess (1) Blood (3) CSF (7) Puncture fluid (1) Deep pus(1)	MLSBc (11) MLSBi (1)	*erm*B (12)	Tn*1545* (11) Tn*6002* (1)
	2307 (1)		2014	I	I	S	R	Blood	MLSBc	*erm*B	Tn*1545*
	16,235 (1)	Portugal^19F^- 21, DLV ST177	2016	I	S	R	R	Blood	MLSBc	*erm*B	Tn*1545*
3	1220 (3)		2013 (1)	S (3)	S (3)	I (1)	R (2)	Blood (2)	MLSBc	*erm*B (2)	Tn*6002* (2)
(12)			2016 (1)			S (2)	S(1)	Deep pus (1)	(2)		
			2018 (1)								
	16,168 (1)		2018	S	S	R	R (1)	Blood	MLSBc	*erm*B	Tn*1545*
	180 (4)		2012	S (4)	S (4)	S (4)	S (4)	Blood (2)			
			2014					Deep pus (1)			
			2015					Abscess (1)			
			2017								
	260 (2)		2016 (2)	S (2)	S (2)	S (2)	S (2)	Blood (2)			
	505 (2)		2014	S (2)	S (2)	S (2)	S (2)	Blood (1)			
			2018					Puncture fluid (1)			
6B	386 (5)	Poland^6B^-	2013 (1)	I (4)	S (5)	R (4)	R (5)	Blood (2)	MLSBc	*erm*B (4)	Tn*1545* (4)
(8)		20, DLV	2016 (1)	S (1)		S (1)		CSF (2)	(4)		Tn*6002* (1)
		ST315	2017 (1)					Deep pus (1)	MLSBi		
			2018 (2)						(1)		
	9194 (1)		2013	S	S	R	R	Blood	MLSBc	*erm*B	Tn*1545*
	16,104 (1)		2013	I	S	R	R	Ascite fluid	MLSBc	*erm*B	Tn*1545*
	16,106 (1)		2015	I	S	S	S	Puncture fluid			
6A (8)	2105 (2)	Sweden^15A^-25, SLV ST63	2016 (1) 2018 (1)	I (2)	S (2)	R (2)	R(2)	Blood (1) CSF (1)	MLSBc (2)	*erm*B (2)	Tn*6003* (2)
	2467 (1)		2015	I	S	S	R	Blood	MLSBi	*erm*B	Tn*6002*
	5679 (1)		2013	I	S	S	R	Blood	MLSBi	*erm*B + *mef*E	Tn*2010*
	16,103 (1)		2014	I	S	S	R	Blood	M	*mef*E	Tn*2009*
	16,105 (1)		2013	I	S	S	R	CSF	M	*mef*E	Tn*2009*
	172 (2)		2017	I (1)	S (2)	S (2)	S (2)	Blood			
			2018	S (1)				CSF			
9 V (6)	156 (2)	Spain^9V^-3 ST156	2012 2015	I (2)	S (2)	R (1) (S1)	R(1) S (1)	Blood (2)	MLSBi	*erm*B	Tn*1545*
	838 (2)	Spain^9V^-3, SLV ST156	2013	R (1)	S (2)	R (1)	R (1)	Blood (1)	MLSBc	*erm*B	Tn*1545*
			2018	I (1)		S (1)	S (1)	CSF (1)			
	280 (2)		2013 2016	S (2)	S (2)	S(2)	S (2)	CSF (2)			
18C	1381 (1)		2016	I	S	R	R	CSF	MLSBc	*erm*B	Tn*1545*
(6)	280 (1)		2018	S	S	S	R	CSF	M	*mef*E	Tn*2009*
	1233 (3)		2013 2014 2015	S(3)	S (3)	S (3)	S (3)	Blood (1) Puncture fluid (1) CSF (1)			
	241 (1)		2016	S	S	S	S	Blood			
19A (5)	3772 (5)	Denmark^14^- 32 DLV ST230	2012 (1) 2016 (2) 2017 (1) 2018 (1)	I (4) R (1)	I (1) S (4)	R (3) I (1) S (1)	R (4) I (1)	Blood (5)	MLSBc (1) MLSBi (3)	*erm*B (4)	Tn*1545* (4)
23F (4)	4003 (3)	Spain^23F-^_-_1, SLV ST81	2014 (1) 2015 (1) 2016 (1)	I (3)	I (2) S (1)	S (3)	R (3)	Blood (2) CSF (1)	MLSBc (3)	*erm*B (3)	Tn*1545* (3)
	81 (1)	Spain^23F^-1 ST81	2012	I	S	R	R	Blood	MLSBc	*erm*B	Tn*1545*
9A (3)	6521 (3)	Spain^9 V^-3 DLV ST156	2012 (1) 2013 (1) 2014 (1)	I (2) R (1)	S (3)	R (3)	R (3)	Blood (3)	MLSBc (3)	*erm*B (3)	Tn*1545* (3)
9 N (3)	6359 (1)		2018	I	S	R	R	Pleural fluid	MLSBc	*erm*B	Tn*1545*
	8809 (1)		2017	I	S	S	S	CSF			
	517 (1)		2018	S	S	R	R	Blood	MLSBc	*erm*B	Tn*1545*
35B (3)	558 (3)	Utah^35B^24, SLV ST377	2013 (1) 2015 (2)	I (3)	S (2) I (1)	S (3)	R (2) S(1)	Blood (2) CSF (1)	M (2)	*mef*E (2)	Tn*2009* (2)
17F (3)	4847 (1) 13,010 (2)		2013 2016 2018	S S I	S S S	S S S	R S S	Blood Blood Blood			
16F (2)	383 3551		2016 2018	S	S	S	S S	Abscess CSF			
4 (2)	17,458 205	Sweeden^4^- 38 ST205	2015 2016	S	S	S	S S	Puncture fluid Blood			
1 (1)	17,380	Sweden^1^- 28, DLV ST306	2014	S	S	S	S	Blood			
24F (1)	16,415	Denmark^14^ −32, DLV ST230	2018	S	S	R	R	Puncture fluid	MLSBc	*erm*B	Tn*1545*
34 (1)	2601		2015	S	S	S	S	CSF			
13 (1)	2187		2013	S	S	S	S	CSF			
35F (1)	15,108		2016	I	S	S	S	CSF			
7F (1)	17,442	Denmark^12^ ^F^-24, DLV ST218	2014	S	S	S	S	Blood			
7C (1)	1201		2014	S	S	S	S	CSF			
6C (1)	386	Poland^6B^- 20, DLV ST315	2014	I	S	R	R	Abscess	MLSBc	*erm*B	Tn*6002*

^a^(): number of isolates

^b^*PeniG* penicillin G

^c^*Ctx* cefotaxim

^d^*Tet* tetracycline

^e^*Ery* erythromycin

^f^*CSF* cerebrospinal fluid

^g^*S* susceptibility

^h^*I* intermediate

^i^*R* resistance

^j^*SLV* Single locus variant

^k^*DLV* double locus variant

### Serotype distribution and vaccine coverage rates

**Fig. 1:**
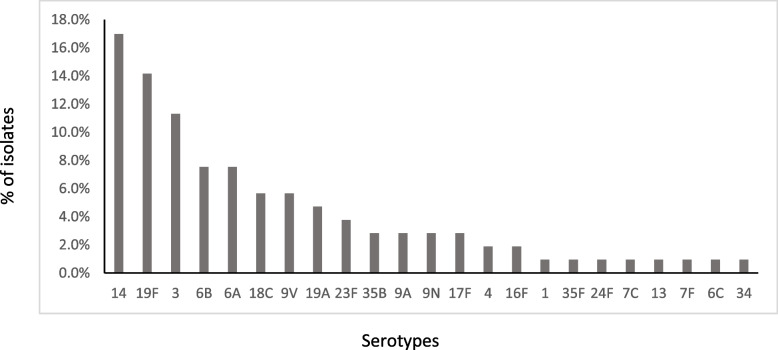
Serotype distribution of invasive *S. pneumoniae* isolates

**Fig. 2:**
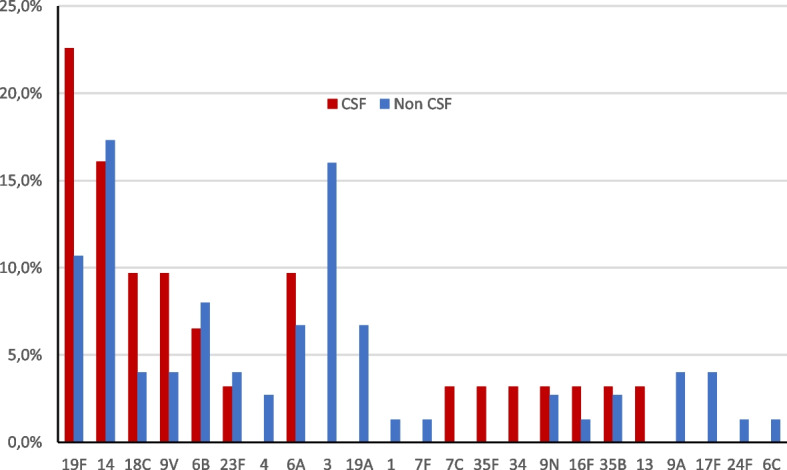
Serotype distribution between meningitis and non-meningitis invasive pneumococcal Disease

We identified 23 different serotypes for 106 invasive *S. pneumoniae* isolates. The most common serotypes were 14 (17%), 19F (14.1%), 3 (11.3%), 6B and 6A (7.6% for each), 18C and 9 V (5.7% for each), 19A (4.7%), 23F (3.8%), and 35B, 9A, and 9 N (2.8% for each) (Table 2, Fig. 1). Serotypes 19F (22.6%), 14 (16.1%), 18C (9.7%), 9 V (9.7%), 6B (6.5%), and 6A (6.5%) were the most common in meningitis cases. Serotypes 14 (17.3%), 3 (16%), 19F (10.7%), 6B (8%), 6A (6.7%), and 19A (6.7%) were the most frequent serotypes in non-meningitis cases, particularly in bacteremia (Fig. 2). Potential immunization coverage rates for pneumococcal conjugate vaccines PCV7, PCV10, and PCV13 were 55.7, 57.5, and 81.1%, respectively.

Stratifying our population by age groups showed that PCV10 and PCV13 coverage was 77.5 and 95% among children < 5 years old, respectively; 66.6 and 88.9% among older children, 5–17 years, respectively; and 43.13 and 70.6% among those ≥18 years of age, respectively.

### Multilocus sequence typing (MLST)

**Fig. 3:**
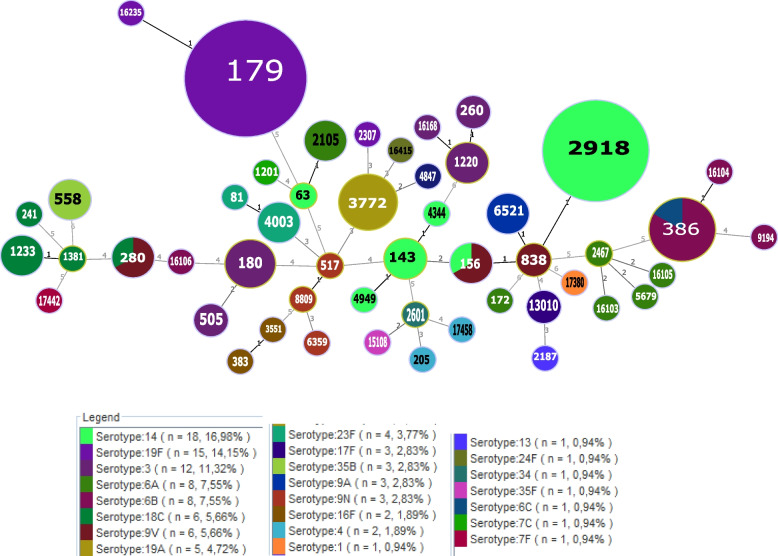
Minimal spanning4 tree of MLST for invasive *S. pneumoniae* iso lates over the 7 –year period. Each circle represents an ST. The area of each circle corresponds to the number of isolates. The numbering shown between the connected nodes indicates the number of loci between the MLST profiles

The 106 invasive pneumococcal disease (IPD) isolates included 50 STs (SID = 0.965, 95% CI = 0.949-0.981). Of these, 18 STs were associated with more than one isolate, and 32 STs were identified with one single isolate. The most frequent STs were ST179 (*n* = 13), ST2918 (*n* = 11), ST386 (*n* = 6), and ST3772 (*n* = 5). We recognized ten STs that were detected for the first time (ST16103, ST16104, ST16105, ST16106, ST16168, ST16235, ST16415, ST17442, ST17458, and ST17380) (Table 2, Fig. 3). We note a significant association of sequence type distribution with the capsular type (*P* = 0.001); as ST179-19F, ST2918-14, ST3772-19A, and ST386- 6B/C (Table 2).

A total of 46 invasive isolates were indistinguishable from or had a single locus variant (SLV) compared to seven international clones recognized by the Pneumococcal Molecular Epidemiology Network (PMEN; http://www.pneumogen.net/pmen/). Serotype switching events were detected in three groups ST156 (9 V and 14), ST280 (9 V and 18C), and ST386 (6B and 6C).

## Discussion

*S. pneumoniae* remains one of the major human pathogens, which can cause various diseases. In developing countries, case fatality rates for invasive pneumococcal diseases remain high, reaching up to 20% for sepsis and 50% for meningitis [2]. In our study, 53.8 and 29.2% of invasive *S. pneumoniae* isolates were from blood and cerebrospinal fluid, respectively. The most common serotypes were 14, 19F, 3, 6A, 6B, 18C, 19A, 9 V, and 23F, accounting for 77.4% of all isolates. Our findings show that *S. pneumoniae* serotypes 19A and 3 that cause IPD were in non-meningitis cases. Serotype 19A was more frequently reported in non-meningitis cases than meningitis [29]. Nevertheless, serotype 3 was observed with or without meningitis [3]. The frequencies of serotypes included in PCV7 (55.7%), PCV10 (57.5%), and PCV13 (81.1%) suggest that the 13-valent PCV would be a potentially useful vaccine in Tunisia. In several European countries, before vaccine implementation, the most common serotypes causing invasive pneumococcal diseases were 14, 6B, 19F, and 23F. However, since the introduction of PCV7, serotypes 1, 3, 6A, 7F, and 19A have become the leading cause among the IPD isolates [8]. Our finding was consistent with data previously reported from studies in Singapore, South Korea, and China that showed an increase in serotype 19A among IPD children before the introduction of PCV7 [30–32].

Genotyping data using MLST showed high diversity among our isolates. Of 50 different STs, ten clonal complexes and 32 singletons were identified. A total of 43.4% of invasive isolates were closely related to seven of the 43 PMEN clones. These clones were Spain^9V^-3-ST156 (*n* = 16), Portugal^19F^-21-ST177 (*n* = 13), Netherlands^3^-31-ST180 (*n* = 4), Spain^23F^-1-ST81 (n = 4), Sweden^15A^-25-ST63 (n = 4), Utah^35B^-24-ST377 (*n* = 3), Colombia^23F^-26-ST338 (n = 1), and Sweeden^4^-38-ST205 (n = 1). As reported previously, we showed high correlations between serotypes and STs (*P* < 0.001). The dominant STs, ST179 (14.3%), ST2918 (12.1%), ST386 (5.5%), ST3772 (5.5%), and ST180 (4.4%) were related to serotypes 19F, 14, 6B, 19A and 3, respectively. It is noticeable that serotypes 14 and 9 V were included in the Spain^9V^-ST156 clone, which has been one of the most successful pneumococcal clones disseminated worldwide before the introduction of PCV-7 [33].

As observed in some countries in the pre-PCV era, the 19A serotype was identified in our population. Nevertheless, the emergence of the 19A serotype was observed worldwide in the post-PCV7 era, particularly in North America and many Asian countries. Mahjoub-Messai reported a clonal expansion of the preexisting penicillin-intermediate ST276 in serotype 19A isolates collected before and after beginning PCV7 vaccination in French children [34]. In Norway, ST3772 and ST276 were detected in 2009, after the introduction of PCV7. ST3772 and ST276 differed only by one locus, *ddl*. ST276 a single-locus variant of the Denmark^14^-32-ST230 clone has been identified in the United States and in southern Europe [35, 36]. Of note that *S. pneumonia* 19A isolates were genetically homogenous and assigned to ST3772. The homogeneous genetic background of 19A and 19F has been reported in the study from multicenter surveillance in China between 2005 and 2011 [31].

We reported for the first time a 6A serotype with ST2105, which was SLV of Sweden^15A^-25. Based on the PubMLST database, ST2105 was previously reported only in 15A and 19F serotypes.

Among eight isolates of serotype 6B *S. pneumoniae*, five belonged to ST386, which has already been described as related to the serotype 6B genetic background of the international Poland^6B^-ST315 clone. In addition, we note that the erythromycin-resistant serotype 6C *S. pneumoniae* isolate was ST386. The presence of serotypes 6B and 6C for ST386 suggest a potential capsular switch event. Janoir et al. (2014) reported a clonal expansion of macrolide-resistant ST386 within pneumococcal serotype 6C in France [37]. In addition, ST386 showed dramatically increased from 5% of all STs in the early PCV7 period to 77% during the PCV13 period (2010–2011). The emergence of serotype 6C-CC386 lineage was also reported in Brazil after the universal use of PCV [38].

Isolates of serotype 6A were more genetically diverse compared to the other serotypes and were associated with distinct STs including two novel STs. The same diversity was reported by Nurse-Lucas et al. (2016) for the serotypes 19F and 23F [10]. However, our findings showed that 19F and 23F serotypes were allied to three and two STs, respectively.

The sequence types ST179, ST3772, and ST2918 were most frequently associated with erythromycin resistance in Tunisia [39]. Reinert et al. (2005) reported the distribution of sequence types among 82 macrolide-resistant pneumococcal isolates from 11 centers in seven European countries. Twenty-three different MLST types were determined including 19 known STs [40]. Four major antimicrobial-resistant Spanish clones of *S. pneumoniae* were identified, including Spain^23F^-1 and Spain^9V^-3. These clones were described in the early 1980s, and they were the most widespread clones in the world. The clone Spain^9V^-3-ST156 and the ST143 appear to be responsible for the increase in antibiotic resistance observed in 2002 in Poland [41]. The Spain^9V^-ST156 clonal complex was the most prevalent clone in invasive penicillin-non-susceptible *S. pneumoniae* isolates recovered in Poland between 2003 and 2005. In addition, macrolide resistance in France is caused particularly by common ST, ST81, and ST143 [33, 40]. Neves et al. (2018) reported that the serotype 14 variant of ST156 was predominant in the pre-PCV10/13 period, but it was not detected in the post-PCV10/13 period [38]. In our study, three sequence types have been identified for serotype 14 *S. pneumoniae* isolates: ST2918, ST143, and ST4949. All these STs are related as single or double locus variants of ST156 and ST838. It is noticeable that ST2918 is rarely described in the world; only two serotype 14 *S. pneumoniae* isolates with ST2918 have been deposited so far on the PubMLST public databases. Recently, Vincent et al. (2019) reported two original cases of neonatal serotype 14 *S. pneumoniae* isolates belonging to ST2918 [42]. Over the past three decades, the prevalence of *S. pneumoniae* with reduced susceptibility to penicillin has increased substantially worldwide. Our results show that penicillin resistance is associated with serotypes 14, 19F, 19A, 9A, and 9 V. High doses of β-lactams or treatment with macrolides are used as alternative approaches to overcome these PNSPs. Unfortunately, the extensive use of macrolide may be linked to increasing macrolide resistance in many countries [5, 6, 43]. In Tunisia, resistance to macrolides was very high with a rate of around 70% [13, 15, 16, 39]. In our study, resistance rates to the tested antibiotics of invasive pneumococcal isolates were high, especially for erythromycin (62.3%) and tetracycline (33%). Erythromycin resistance is linked to serotypes 6A and 6B. Recently, analyses of data from 559 epidemiological studies across 104 countries revealed the greatest prevalence of non-susceptibility and resistance for penicillin, macrolides, third-generation cephalosporins, and tetracycline in southeast Asia, east Asia, and Oceania. The high rate of non-susceptibility of macrolides was also reported in high-income Asia-Pacific countries [44].

In our study, the mechanism of resistance to macrolides showed that the enzymatic modification of the MLS binding site was predominant. We noted that 89.4% of the isolates were MLSB phenotype while the efflux mechanism was only 10.6%. Our outcome also confirmed that the majority of isolates (77.3%) exhibited cMLSB phenotype, with iMLSB and M phenotypes observed in 12.1 and 10.6% of the isolates, respectively. cMLSB phenotype is predominant in most European countries and particularly in France, Spain, and Switzerland, whereas the M phenotype predominates in North America, England, and Germany [36]. According to recent studies, the cMLSB phenotype was the most frequent in Tunisia (75.5%) [45]. The macrolide-resistant phenotypes were genotypically confirmed by the presence of *erm*B (87.5%) and *mef*E (10.9%) genes. The high prevalence of the *erm*B gene has also been described in France (90%) and in Belgium (91.5%). In contrast, in the United States and Canada, the *mef* gene was the most prevalent, exceeding 50% [46]. The widespread dissemination of antibiotic resistance among pneumococci is associated with mobile genetic elements, such as transposons. In our study, the transposon Tn*1545* is responsible for the propagation of most resistance to tetracycline and erythromycin (76.6%).

In conclusion, the common serotypes of invasive *S. pneumoniae* were 14, 19F, and 3 before the introduction of PCV-10 in the national immunization program in April 2019. The most common serotypes in meningitis cases were 19F, 14, 18C, 9 V, 6B, and 6A. For non-meningitis cases, the most frequent serotypes were 14, 3, 19F, 6B, 6A, and 19A. We showed high rates of non-susceptible isolates for penicillin, erythromycin, and tetracycline in the pre-PCV period. Thus, 87.9% of macrolide-resistant invasive isolates harbored the *erm*B gene. Population structure analysis showed that most serotypes (19F, 14, 6B, 19A, and 23F) are grouped in different clones and CCs as previously described in Tunisia and in European areas, such as Italy, Spain, France, and Poland. The capsular switching event was infrequent in invasive isolates, and it played a minor role in this population. However, this event may be an important source of new variants that may increase in the post-PCVs period. To better evaluate the genetic evolution and to monitor the antimicrobial-resistant and expanding clones, whole genome sequencing will be a necessary tool to use in future studies.

## Data Availability

The new alleles spi680 and gdh708 were deposited at the National Center for Biotechnology Information (NCBI) with the following GenBank accession numbers MZ338258 (https://www.ncbi.nlm.nih.gov/nuccore/MZ338258) and MZ338259 (https://www.ncbi.nlm.nih.gov/nuccore/MZ338259), respectively. We declare that the data supporting the conclusions of this article are available from the corresponding author upon reasonable request.

## References

[1] Shetty AK, Maldonado YA (2013). Current trends in *Streptococcus pneumoniae* infections and their treatment. Curr Pediatr Rep.

[2] World Health Organization (2018). Surveillance vaccine preventable. Pneumococcus.

[3] Torné AN, Dias JG, Quinten C, Hruba F, Busana MC, Lopalco PL (2014). European enhanced surveillance of invasive pneumococcal disease in 2010: data from 26 European countries in the post-heptavalent conjugate vaccine era. Vaccine..

[4] Kaplan SL, Mason EO (1998). Management of Infections due to antibiotic-resistant *Streptococcus pneumoniae*. Clin Microbiol Rev.

[5] Schroeder MR, Stephens DS. Macrolide resistance in *Streptococcus pneumoniae*. Front Cell Infect Microbiol. 2016;6. 10.3389/fcimb.2016.00098. 10.3389/fcimb.2016.00098 PMC5030221 27709102

[6] Leclercq R, Courvalin P (2002). Resistance to macrolides and related antibiotics in *Streptococcus pneumoniae*. Antimicrob Agents Chemother.

[7] Ganaie F, Saad JS, McGee L, van Tonder AJ, Bentley SD, Lo SW (2020). A new pneumococcal capsule type, 10D, is the 100th serotype and has a large *cps* fragment from an Oral *streptococcus*. McDaniel LS, editor. mBio.

[8] Johnson HL, Deloria-Knoll M, Levine OS, Stoszek SK, Freimanis Hance L, Reithinger R (2010). Systematic evaluation of serotypes causing invasive pneumococcal disease among children under five: the pneumococcal global serotype project. Cohen J, editor. PLoS Med.

[9] Oligbu G, Fry NK, Ladhani SN (2019). The pneumococcus and its critical role in public health. Methods Mol Biol.

[10] Nurse-Lucas M, McGee L, Hawkins PA, Swanston WH, Akpaka PE (2016). Serotypes and genotypes of *Streptococcus pneumoniae* isolates from Trinidad and Tobago. Int J Infect Dis.

[11] Scelfo C, Menzella F, Fontana M, Ghidoni G, Galeone C, Facciolongo NC (2021). Pneumonia and invasive pneumococcal diseases: the role of pneumococcal conjugate vaccine in the era of multi-drug resistance. Vaccines..

[12] Ziane H, Manageiro V, Ferreira E, Moura IB, Bektache S, Tazir M, et al. Serotypes and antibiotic susceptibility of *Streptococcus pneumoniae* isolates from invasive pneumococcal disease and asymptomatic carriage in a pre-vaccination period, in Algeria. Front Microbiol. 2016;7. 10.3389/fmicb.2016.00803. 10.3389/fmicb.2016.00803 PMC4905970 27379023

[13] Rachdi M, Boutiba-Ben Boubaker I, Moalla S, Smaoui H, Hammami A, Kechrid A (2008). Phenotypic and genotypic characterization of macrolide resistant *Streptococcus pneumoniae* in Tunisia. Pathol Biol.

[14] Raddaoui A, Tanfous FB, Chebbi Y, Achour W, Baaboura R, Benhassen A (2018). High prevalence of multidrug-resistant international clones among macrolide-resistant *Streptococcus pneumoniae* isolates in immunocompromised patients in Tunisia. Int J Antimicrob Agents.

[15] Ktari S, Jmal I, Mroua M, Maalej S, Ben Ayed NE, Mnif B (2017). Serotype distribution and antibiotic susceptibility of *Streptococcus pneumoniae* strains in the south of Tunisia: a five-year study (2012–2016) of pediatric and adult populations. Int J Infect Dis.

[16] Ben Ayed N, Ktari S, Mezghani S, Mnif B, Mahjoubi F, Hammami A (2022). Relationship between serotypes and antimicrobial nonsusceptibility of *Streptococcus pneumoniae* clinical isolates in Tunisia. Microb Drug Resist.

[17] Huang S, Liu X, Lao W, Zeng S, Liang H, Zhong R (2015). Serotype distribution and antibiotic resistance of *Streptococcus pneumoniae* isolates collected at a Chinese hospital from 2011 to 2013. BMC Infect Dis.

[18] Mukerji R, Briles DE (2020). New strategy is needed to prevent pneumococcal meningitis. Pediatr Infect Dis J.

[19] Rayner RE, Savill J, Hafner LM, Huygens F (2015). Genotyping *Streptococcus pneumoniae*. Future Microbiol.

[20] Horna G, Molero ML, Benites L, Roman S, Carbajal L, Mercado E (2016). Oxacillin disk diffusion testing for the prediction of penicillin resistance in *Streptococcus pneumoniae*. Rev Panam Salud Publica.

[21] Wierzbowski AK, Karlowsky JA, Adam HJ, Nichol KA, Hoban DJ, Zhanel GG (2014). Evolution and molecular characterization of macrolide-resistant *Streptococcus pneumoniae* in Canada between 1998 and 2008. J Antimicrob Chemother.

[22] Sutcliffe J, Grebe T, Tait-Kamradt A, Wondrack L (1996). Detection of erythromycin-resistant determinants by PCR. Antimicrob Agents Chemother.

[23] Del Grosso M, Iannelli F, Messina C, Santagati M, Petrosillo N, Stefani S (2002). Macrolide efflux genes *mef* (a) and *mef* (E) are carried by different genetic elements in *Streptococcus pneumoniae*. J Clin Microbiol.

[24] Talebi M, Azadegan A, Sadeghi J, Ahmadi A, Ghanei M, Katouli M (2016). Determination of characteristics of erythromycin resistant *Streptococcus pneumoniae* with preferred PCV usage in Iran. PLoS One.

[25] Aminov R, Chee-Sanford JC, Garrigues N, Mehboob A, Mackie RI (2004). Detection of tetracycline resistance genes by PCR methods. Methods Mol Biol.

[26] Pai R, Gertz RE, Beall B (2006). Sequential multiplex PCR approach for determining capsular serotypes of *Streptococcus pneumoniae* isolates. J Clin Microbiol.

[27] Ziane H, Manageiro V, Ferreira E, Bektache S, Tazir M, Caniça M (2015). Capsular typing of Streptococcus pneumoniae isolated in an Algerian hospital using a new multiplex PCR-based scheme. J Microbiol Methods.

[28] Francisco AP, Vaz C, Monteiro PT, Melo-Cristino J, Ramirez M, Carriço JA (2012). PHYLOViZ: phylogenetic inference and data visualization for sequence based typing methods. BMC Bioinformatics.

[29] Castañeda E, Agudelo CI, De Antonio R, Rosselli D, Calderón C, Ortega-Barria E (2012). *Streptococcus pneumoniae* serotype 19A in Latin America and the Caribbean: a systematic review and meta-analysis, 1990–2010. BMC Infect Dis.

[30] Choi EH, Kim SH, Eun BW, Kim SJ, Kim NH, Lee J (2008). *Streptococcus pneumoniae* serotype 19A in children, South Korea. Emerg Infect Dis.

[31] Zhao C, Zhang F, Chu Y, Liu Y, Cao B, Chen M (2013). Phenotypic and genotypic characteristic of invasive pneumococcal isolates from both children and adult patients from a multicenter surveillance in China 2005–2011. PLoS One.

[32] Thoon KC, Chong CY, Tee NWS (2012). Early impact of pneumococcal conjugate vaccine on invasive pneumococcal disease in Singapore children, 2005 through 2010. Int J Infect Dis.

[33] Sadowy E, Kuch A, Gniadkowski M, Hryniewicz W (2010). Expansion and evolution of the *Streptococcus pneumoniae* Spain ^9V^ -ST156 clonal complex in Poland. Antimicrob Agents Chemother.

[34] Mahjoub-Messai F, Doit C, Koeck J-L, Billard T, Evrard B, Bidet P (2009). Population snapshot of *Streptococcus pneumoniae* serotype 19A isolates before and after introduction of seven-Valent pneumococcal vaccination for French children. J Clin Microbiol.

[35] Siira L, Vestrheim DF, Winje BA, Caugant DA, Steens A (2020). Antimicrobial susceptibility and clonality of *Streptococcus pneumoniae* isolates recovered from invasive disease cases during a period with changes in pneumococcal childhood vaccination, Norway, 2004–2016. Vaccine..

[36] Vestrheim DF, Steinbakk M, Aaberge IS, Caugant DA (2012). Postvaccination increase in serotype 19A pneumococcal disease in Norway is driven by expansion of penicillin-susceptible strains of the ST199 complex. Clin Vaccine Immunol.

[37] Janoir C, Cohen R, Levy C, Bingen E, Gutmann L, Varon E (2014). Clonal expansion of the macrolide resistant ST386 within pneumococcal serotype 6C in France. PLoS One.

[38] Neves FPG, Cardoso NT, Souza ARV, Snyder RE, Marlow MM, Pinto TCA (2018). Population structure of *Streptococcus pneumoniae* colonizing children before and after universal use of pneumococcal conjugate vaccines in Brazil: emergence and expansion of the MDR serotype 6C-CC386 lineage. J Antimicrob Chemother.

[39] Midouni Ayadi B, Mehiri E, Draoui H, Ghariani A, Essalah L, Raoult D (2020). Phenotypic and molecular characterization of macrolide resistance mechanisms among *Streptococcus pneumoniae* isolated in Tunisia. J Med Microbiol.

[40] Reinert RR, Ringelstein A, van der Linden M, Cil MY, Al-Lahham A, Schmitz F-J (2005). Molecular epidemiology of macrolide-resistant *Streptococcus pneumoniae* isolates in Europe. J Clin Microbiol.

[41] Korona-Glowniak I, Maj M, Siwiec R, Niedzielski A, Malm A (2016). Molecular epidemiology of *Streptococcus pneumoniae* isolates from children with recurrent upper respiratory tract infections. Ho PL, editor. PLoS One.

[42] Vincent A, Bonacorsi S, Varon E, Dauger S, Levy M (2019). Serotype 14 pneumococcal bacteremia: from one neonate to another in a pediatric intensive care unit. Infect Control Hosp Epidemiol.

[43] Akdoğan Kittana FN, Mustak IB, Hascelik G, Saricam S, Gurler N, Diker KS (2019). Erythromycin-resistant *Streptococcus pneumoniae*: phenotypes, genotypes, transposons and pneumococcal vaccine coverage rates. J Med Microbiol.

[44] Andrejko K, Ratnasiri B, Hausdorff WP, Laxminarayan R, Lewnard JA (2021). Antimicrobial resistance in paediatric *Streptococcus pneumoniae* isolates amid global implementation of pneumococcal conjugate vaccines: a systematic review and meta-regression analysis. Lancet Microbe.

[45] Midouni B, Mehiri E, Ghariani A, Draoui H, Essalah L, Bouzouita I (2019). Genetic diversity of *Streptococcus pneumoniae* in Tunisia. Int J Antimicrob Agents.

[46] Farrell DJ, Morrissey I, Bakker S, Felmingham D (2002). Molecular characterization of macrolide resistance mechanisms among *Streptococcus pneumoniae* and *streptococcus pyogenes* isolated from the PROTEKT 1999-2000 study. J Antimicrob Chemother.

